# A comprehensive multivariate model of biopsychosocial factors associated with opioid misuse and use disorder in a 2017–2018 United States national survey

**DOI:** 10.1186/s12889-020-09856-2

**Published:** 2020-11-18

**Authors:** Francisco A. Montiel Ishino, Philip R. McNab, Tamika Gilreath, Bonita Salmeron, Faustine Williams

**Affiliations:** 1 Division of Intramural Research, National Institute on Minority Health and Health Disparities, National Institutes of Health, 7201 Wisconsin Ave Ste. 533, Bethesda, MD 20814 USA; 2 Center for a Livable Future, Department of Environmental Health & Engineering, Johns Hopkins Bloomberg School of Public Health, 111 Market Place, Suite 840, Baltimore, MD 21202 USA; 3 Transdisciplinary Center for Health Equity Research, College of Education and Human Development, Texas A&M University, 4243 TAMU, 311 Blocker, College Station, TX 77843 USA

**Keywords:** Opioids, Opioid misuse, Opioid use disorder, Biopsychosocial factors, Comprehensive risk

## Abstract

**Background:**

Few studies have comprehensively and contextually examined the relationship of variables associated with opioid use. Our purpose was to fill a critical gap in comprehensive risk models of opioid misuse and use disorder in the United States by identifying the most salient predictors.

**Methods:**

A multivariate logistic regression was used on the 2017 and 2018 National Survey on Drug Use and Health, which included all 50 states and the District of Columbia of the United States. The sample included all noninstitutionalized civilian adults aged 18 and older (*N* = 85,580; weighted *N* = 248,008,986). The outcome of opioid misuse and/or use disorder was based on reported prescription pain reliever and/or heroin use dependence, abuse, or misuse. Biopsychosocial predictors of opioid misuse and use disorder in addition to sociodemographic characteristics and other substance dependence or abuse were examined in our comprehensive model. Biopsychosocial characteristics included socioecological and health indicators. Criminality was the socioecological indicator. Health indicators included self-reported health, private health insurance, psychological distress, and suicidality. Sociodemographic variables included age, sex/gender, race/ethnicity, sexual identity, education, residence, income, and employment status. Substance dependence or abuse included both licit and illicit substances (i.e., nicotine, alcohol, marijuana, cocaine, inhalants, methamphetamine, tranquilizers, stimulants, sedatives).

**Results:**

The comprehensive model found that criminality (adjusted odds ratio [AOR] = 2.58, 95% confidence interval [CI] = 1.98–3.37, *p* < 0.001), self-reported health (i.e., excellent compared to fair/poor [AOR = 3.71, 95% CI = 2.19–6.29, *p* < 0.001], good [AOR = 3.43, 95% CI = 2.20–5.34, p < 0.001], and very good [AOR = 2.75, 95% CI = 1.90–3.98, *p* < 0.001]), no private health insurance (AOR = 2.12, 95% CI = 1.55–2.89, *p* < 0.001), serious psychological distress (AOR = 2.12, 95% CI = 1.55–2.89, *p* < 0.001), suicidality (AOR = 1.58, 95% CI = 1.17–2.14, *p* = 0.004), and other substance dependence or abuse were significant predictors of opioid misuse and/or use disorder. Substances associated were nicotine (AOR = 3.01, 95% CI = 2.30–3.93, *p* < 0.001), alcohol (AOR = 1.40, 95% CI = 1.02–1.92, *p* = 0.038), marijuana (AOR = 2.24, 95% CI = 1.40–3.58, *p* = 0.001), cocaine (AOR = 3.92, 95% CI = 2.14–7.17, *p* < 0.001), methamphetamine (AOR = 3.32, 95% CI = 1.96–5.64, *p* < 0.001), tranquilizers (AOR = 16.72, 95% CI = 9.75–28.65, *p* < 0.001), and stimulants (AOR = 2.45, 95% CI = 1.03–5.87, *p* = 0.044).

**Conclusions:**

Biopsychosocial characteristics such as socioecological and health indicators, as well as other substance dependence or abuse were stronger predictors of opioid misuse and use disorder than sociodemographic characteristics.

## Background

Estimates indicate that up to 29% of persons misuse prescription pain relievers for chronic pain, [1] and between 8 to 12% develop a use disorder [2, 3]. The United States (US) Department of Health and Human Services declared the opioid crisis a public health emergency in 2017, although the first wave of the epidemic emerged in the 1990s [3]. Opioid related deaths increased 345% between 2001 to 2016 [4]. Subsequently, between July 2016 and September 2017 deaths due to illicit opioid overdose increased by 30%, leading to an emergency declaration in 45 states [4].

Projections indicate that if current prevention and intervention strategies do not change by 2025, the rate of misuse and overdose death will rise by 61% [5]. In response to the epidemic, multiple federal, state, and local agencies have implemented various strategies to address the opioid crisis. Increasing the availability of naloxone—a medication that reverses the effects of an overdose—is projected to reduce opioid-related deaths by approximately 4% according to the most recent projections [6]. Other interventions like reduced prescribing for pain patients and excess opioid management can increase life years and quality-adjusted life years, but overdose deaths would increase among those with opioid dependence due to a move from prescription opioids to heroin [6]. Overall, supply-side prevention strategies are estimated to have minimal impact, preventing only 3.0 to 5.3% of overdose deaths [6].

As current interventions are inadequately addressing the multidimensional and far-reaching nature of the opioid epidemic [5, 6], some scholars have suggested developing more tailored approaches to reach specific, underrepresented populations [7]. Non-Hispanic whites, for instance, have become the primary focus for multiple prevention programs and strategies as they have been found to misuse opioid at greater rates [8–10]. However, multiple racial/ethnic groups have been found to be at differential risk, as well as differentially affected by opioid misuse [8–10].

Opioid misuse and/or use disorder are also linked to other risk factors besides race and ethnicity. Scholl et al. [9] found that younger age was a significant predictor of misuse. The current opioid misuse and/or use disorder literature has also found that race/ethnicity and age become less predictive of misuse when they are considered in the context of other biopsychosocial factors such as sex and gender. For instance, Nicholson and Vincent [11] observed that Black women with lower socioeconomic status had an increased the probability of misuse, while older age, higher educational attainment, and rural residence were associated with a lower probability [11, 12].

Other biopsychosocial factors like criminality and sexual identity, although understudied, have been associated with misuse and/or use disorder [13, 14]. For instance, Pierce et al. [13] found that individuals testing positive for opioid use had higher rates of criminality—though the relationship was strongest for less serious crimes. Sexual minorities, such as those identifying as gay/lesbian or bisexual, have also been reported to be at risk of opioid misuse [14–16]. For instance, Duncan et al. [14] found that, compared to heterosexuals, those identifying as bisexual or gay/lesbian were at 78 and 115% increased odds of misuse, respectively.

General health and access to healthcare have a role in opioid misuse and/or use disorder, but most research has focused on hospitalized subpopulations and physical pain [1, 17, 18], which will not be covered here. The general health and access to healthcare relationship, however, is less clear among noninstitutionalized populations. One particular aspect of healthcare access in the form of health insurance is believed to have a role in opioid misuse. Some studies argue that health insurance companies may facilitate opioid misuse [19], whereas others have observed that an increase in health insurance coverage was linked to a reduction in opioid-related deaths [20]. Mental health is another facet of health for which there is an unclear relationship with opioid misuse and/or use disorder, as specific disorders may influence the association differently. Nevertheless, opioid misuse and/or use disorder has been found to be associated with severe mental illness like depression and anxiety [21, 22], as well as suicidality [22–24].

Concurrent substance use such as nicotine and tobacco dependence [25, 26], alcohol [27], sedatives [28], methamphetamines [29], tranquilizers [30–32], other analgesics [33], and marijuana [34] have been positively associated with opioid misuse and use disorder [34, 35]. Marijuana’s association may be context dependent, as it has a mixed relationship with opioid use, misuse, and use disorder [36]. Polysubstance abuse must be critically assessed in context of opioid use as multiple associations may exist due to the varied effects of synergizing the opioid high. A better understanding of how polysubstance abuse occurs in context of multiple social and environmental factors is critical [28, 29, 37].

We hypothesized that sociodemographic factors, while crucial to the comprehensive risk model, would not be critical predictors, when they were included with socioecological and health factors, or with other substance dependence or abuse. The purpose of this study was to fill in a critical gap in the literature to improve population-level prevention strategies by identifying the most salient predictors of opioid misuse and/or use disorder.

## Methods

While epidemiologic studies have examined the relationship of various risk factors on opioid misuse and use disorder among non-institutionalized populations, comprehensive models are relatively absent. To address the opioid epidemic, we need to identify the risk factors associated with the etiology of misuse to intervene at multiple levels, tailor interventions for specific populations, and prevent the distal events of use disorder like overdose. In response to this need, we comprehensively examined the relationship of opioid misuse and/or use disorder and biopsychosocial characteristics using four domains: (1) sociodemographic factors; (2) socioecological factors (e.g., criminality); (3) health factors (e.g., self-reported general health; mental health, suicidality; access to health services); and (4) other substance dependence or abuse. We took this approach to determine the most salient risk factors for opioid misuse and/or use disorder in a representative, noninstitutionalized US adult sample.

We used multivariate logistic regression on the combined 2017 [38] and 2018 [39] National Survey on Drug Use and Health (NSDUH) to examine the relationship of biopsychosocial characteristics and opioid misuse and/or use disorder. Opioid misuse was characterized as heroin use and/or prescription pain reliever misuse in the past year based on NSDUH definitions [40]. Individuals taking prescribed pain relievers may develop a tolerance to pain relief that can lead to taking the prescription at higher doses and/or more frequently than prescribed, which would constitute misuse [40]. Furthermore, heroin was included with misuse as any opioid creates the same adverse effects as prescription pain relievers, which in turn may develop into opioid use disorder [40]. Use disorder was characterized by heroin use disorder, prescription pain reliever use disorder, or heroin and prescription pain reliever use disorder, as they may not be mutually exclusive in the NSDUH [40]. Biopsychosocial characteristics, as well as sociodemographic and other substance dependence or abuse were tested independently in unadjusted models. Adjusted models were then built using a block entry method to test biopsychosocial characteristics on opioid misuse and/or misuse disorder in the following order: (Model 1) sociodemographic indicators; (Model 2) socioecological indicator; (Model 3) health indicators; and (Model 4) other substance dependence or abuse. All variables were retained as controls and covariates in subsequent models. We accounted for the complex survey design of the NSDUH by the strata and clusters provided, as well as adjusting the analytical weights to account for two years. All analyses were conducted with Stata 16 (StataCorp LLC, College Station, TX). The study received exemption from the Institutional Review Board, as no human participants were involved in this research. The analysis was not pre-registered, and the results should be considered exploratory.

### Sociodemographic variables and factors

Five age categories were used: (1) 18 to 25; (2) 26 to 34; (3) 35 to 49; (4) 50 to 64; and (5) 65 and older. The binary category of male and female was used for sex/gender. Race/ethnicity was divided into seven categories: (1) non-Hispanic white; (2) non-Hispanic Black/African American; (3) non-Hispanic Native American/Alaska Native; (4) non-Hispanic Native Hawaiian/other Pacific Islander; (5) non-Hispanic Asian; (6) non-Hispanic more than one race; and (7) Hispanic. Sexual identity had three categories: (1) heterosexual; (2) gay/lesbian; and (3) bisexual. Place of residence was based on 2009 Core-Based Statistical Areas (CBSAs) defined by the Office of Management and Budget [41]: (1) CBSA with 1 million or more persons; (2) CBSA with fewer than 1 million persons; and (3) segment not in a CBSA. Total family income was divided into four categories: (1) less than $20,000; (2) $20,000 to $49,999; (3) $50,000 to $74,999; and (4) $75,000 or more. Employment status was divided into five categories: (1) full−/part-time job; (2) unemployed; (3) retired; (4) disabled; and (5) other which included keeping house full time and in school/training. Educational attainment was divided into four categories: (1) less than high school; (2) high school graduate; (3) some college/associate’s degree; and (4) college graduate.

### Socioecological factors

The criminality variable was based on if the participant had been arrested and booked for breaking the law, excluding minor traffic violations. Booked was defined as being taken into custody and processed by the legal system, even if the participant was later released.

### Health factors

Health factors included overall perceived health, having access to private health insurance, and mental health indicators. Overall self-reported health was categorized as (1) excellent, (2) very good, (3) good, and (4) fair/poor. The private health insurance category was based on if respondent had obtained it through (1) employment by paying premiums to an insurance company; (2) the Health Insurance Marketplace; or (3) a health maintenance organization (HMO), fee-for-service plans, or single-service plans. The mental health indicators were severe psychological distress and suicidality. A severe psychological distress indicator within the past year was based on responses from past-month Kessler-6 (K6) items and the worst month in the past-year K6 items. K6 items are from a screening instrument for nonspecific psychological distress developed by Furukawa, Kessler, Slade, and Andrews, [42] and Kessler et al. [43] Suicidality was assessed if at any time in the past year a participant had seriously thought about trying to commit suicide.

### Substance misuse, dependence, and/or abuse factors

The outcome of opioid misuse and/or use disorder was defined as misuse and/or dependence or abuse of prescription pain relievers and/or heroin use in the past year. Opioid use disorder was classified using the Diagnostic and Statistical Manual of Mental Disorders, 4th edition (DSM-IV) criteria for dependence or abuse criteria based on heroin use disorder, prescription pain reliever use disorder, or heroin and prescription pain reliever use disorder in the past year based on NSDUH methodology and terminology [See https://www.samhsa.gov/data/sites/default/files/cbhsq-reports/NSDUHMethodsSummDefs2018/NSDUHMethodsSummDefs2018.pdf]. While opioid substance use disorder was classified under the DSM-V, the NSDUH used the DSM-IV criteria of dependence or abuse, as such we opted to use the DSM-V terminology [15, 18]. Nicotine dependence in the past month was assessed using Nicotine Dependence Syndrome Scale scores and the Fagerstrom Test of Nicotine Dependence scale in the past month. Alcohol dependence and abuse in the last year was also ascertained. Dependence and abuse in the past year were also determined for marijuana, cocaine, hallucinogens, inhalants, methamphetamine, tranquilizers, stimulants (i.e., independent of methamphetamine), and sedatives [44].

### Statistical analysis

We performed descriptive analyses to detail the characteristics of NSDUH sample participants. We checked the data for normality of the residuals, homoscedasticity, multicollinearity, outliers and influence. After the data were found to be adequate for the logistic regression model, four weighted multivariate models were built using Stata survey procedure. All models were weighted and accounted for clustering and stratification of the complex survey design. All findings are reported in odds ratios (ORs) or adjusted odds ratios (AORs) using a 95% confidence interval (CI) and *p*-value for significance criteria.

## Results

### Sample characteristics

The sample consisted of 85,580 individuals (weighted *N* = 248,008,986) over the age of 18. Male and female participants were represented about equally—48% male (weighted *N* = 119,711,438) and 52% female (weighted *N* = 119,711,438). The majority of the weighted sample was non-Hispanic white (63.6%), resided in a high population density CBSA (54.1%), identified as heterosexual (94.8%), had a family income of $75,000 or more (38.9%), were college graduates (32.1%), were employed (62.7%), had no history of arrest and booking (83.4%), were in very good health (36.1%), had private health insurance (66.6%), had no serious psychological distress in past year (88.6%), and displayed no suicidality (95.7%). See Table 1 for a detailed breakdown of the sample’s characteristics.

**Table 1:** Descriptive characteristics of biopsychical indicators using the 2017–2018 NSDUH (*N* = 85,580; Weighted *N* = 248,008,986)

	*N*	Weighted *N*	%
Age Groups			
18–25 years old	27,477	34,171,330	13.8%
26–34 years old	17,580	39,791,188	16.0%
35–49 years old	22,902	61,084,084	24.6%
50–64 years old	9935	62,285,999	25.1%
65 or older	7686	50,676,385	20.4%
Sex/Gender			
Male	40,156	119,711,438	48.3%
Female	45,424	128,297,548	51.7%
Race/Ethnicity			
Non-Hispanic White	51,704	157,708,305	63.6%
Non-Hispanic Black/African American	10,630	29,520,476	11.9%
Native American/Alaska Native	1220	1,387,749	0.6%
Native Hawaiian/other Pacific Islander	417	939,268	0.4%
Non-Hispanic Asian	4190	14,061,853	5.7%
Non-Hispanic more than one race	2786	4,250,536	1.7%
Hispanic	14,633	40,140,798	16.2%
Area of Residence by Population Density			
Segment in a CBSA > 1 million	36,272	134,292,992	54.1%
Segment in a CBSA < 1 million	42,433	99,166,152	40.0%
Segment not in a CBSA	6875	14,549,842	5.9%
Sexual Identity			
Heterosexual, i.e., straight	77,811	230,292,107	94.8%
Lesbian or gay	1884	4,774,123	2.0%
Bisexual	4204	7,875,005	3.2%
Family Income			
Less than $20,000	16,488	39,520,535	15.9%
$20,000–$49,999	26,460	72,948,368	29.4%
$50,000–$74,999	13,376	38,994,110	15.7%
$75,000 or more	29,256	96,545,973	38.9%
Level of Education			
Less than high school	10,832	30,482,047	12.3%
High school graduate	22,532	61,032,429	24.6%
Some college/associate’s degree	28,608	76,994,245	31.0%
College graduate	23,608	79,500,265	32.1%
Employment Status (past week)			
Employed full/part-time	57,686	153,914,559	62.7%
Unemployed	4840	10,241,227	4.2%
Retired	6329	41,374,848	16.9%
Disabled	3035	11,545,013	4.7%
Other	12,717	28,404,275	11.6%
Ever Arrested and Booked			
No	70,625	205,996,442	83.4%
Yes	14,628	41,013,634	16.6%
Overall Health Status			
Fair/poor	9675	34,313,374	13.8%
Good	23,960	72,114,751	29.1%
Very good	32,368	89,447,218	36.1%
Excellent	19,555	52,070,096	21.0%
Covered by Private Health Insurance			
No	30,721	82,568,583	33.4%
Yes	54,422	164,350,599	66.6%
Serious Psychological Distress Indicator (past year)			
No	72,141	219,851,056	88.6%
Yes	13,439	28,157,930	11.4%
Suicidality (past year)			
No	79,598	235,697,531	95.7%
Yes	5327	10,703,135	4.3%

Of the sample, 865 individuals (weighted *N* = 1,976,471) reported opioid misuse. Other substances that the sample had dependence on or abused were nicotine, alcohol, marijuana, cocaine, inhalants, methamphetamine, tranquilizers, stimulants, hallucinogens, and sedatives. See Table 2 for a complete report of the sample’s substance dependence and abuse profile.

**Table 2:** Descriptive characteristics of substance dependence or abuse from the 2017–2018 NSDUH (*N* = 85,580; Weighted *N* = 248,008,986)

	*N*	Weighted *N*	%
Nicotine dependence (past month)			
No	75,397	221,362,313	89.26%
Yes	10,183	26,646,673	10.74%
Alcohol dependence or abuse (past year)			
No/Unknown	79,239	133,842,026	94.29%
Yes	6341	14,166,959	5.71%
Marijuana dependence or abuse (past year)			
No/Unknown	83,439	244,355,720	98.53%
Yes	2141	36,532,266	1.47%
Cocaine dependence or abuse (past year)			
No/Unknown	85,147	247,063,145	99.62%
Yes	433	945,841	0.38%
Inhalant dependence or abuse (past year)			
No	85,535	247,914,187	99.96%
Yes	45	94,798	0.04%
Methamphetamine dependence or abuse (past year)			
No	85,146	246,985,929	99.59%
Yes	434	1,023,057	0.41%
Tranquilizer dependence or abuse (past year)			
No	85,260	247,362,108	99.74%
Yes	320	646,877	0.26%
Stimulant dependence or abuse (past year)			
No	85,309	247,499,633	99.79%
Yes	271	509,353	0.21%
Sedative dependence or abuse (past year)			
No	85,519	247,855,708	99.94%
Yes	61	153,278	0.06%
Opioid dependence or abuse (past year)			
No	84,715	246,032,515	99.20%
Yes	865	1,976,471	0.80%

### Logistic regression

#### Independent unadjusted models

All sociodemographic and biopsychosocial characteristics, as well as other substance dependence or abuse were tested independently in unadjusted models to examine the relationship of each characteristic on opioid misuse. All characteristics tested with exception of residence at some level were found to be a significant factor predictive of opioid misuse. See Table 3 for all associations.

**Table 3:** Odds ratios, 95% confidence intervals, and *p*-values of independent biopsychosocial indicators and other substance dependence or abuse on opioid misuse: 2017–2018 National Survey on Drug Use and Health

	OR	95% CI		*p*-value
		Lower	Upper	
Age				
18–25 years old	**6.55**	**3.10**	**13.83**	**0.000**
26–34 years old	**7.97**	**3.77**	**16.84**	**0.000**
35–49 years old	**4.95**	**2.33**	**10.52**	**0.000**
50–64 years old	**4.86**	**2.35**	**10.04**	**0.000**
65 years and older	ref.	–	–	–
Sex/Gender				
Male	**1.43**	**1.14**	**1.80**	**0.003**
Female	ref.	–	–	–
Race/Ethnicity				
Non-Hispanic White	**5.15**	**2.31**	**11.46**	**0.000**
Non-Hispanic Black/African American	**3.95**	**1.60**	**9.77**	**0.004**
Native American/Alaska Native	**8.64**	**3.28**	**22.75**	**0.000**
Native Hawaiian/Pacific Islander	3.39	0.65	17.61	0.142
Non-Hispanic more than one race	**7.48**	**2.84**	**19.65**	**0.000**
Hispanic	**3.18**	**1.42**	**7.12**	**0.006**
Non-Hispanic Asian	ref.	–	–	–
Sexual Identity				
Lesbian or gay	1.21	0.70	2.08	0.484
Bisexual	**2.70**	**1.89**	**3.84**	**0.000**
Heterosexual, i.e., straight	ref.	–	–	–
Educational attainment				
Less than high school	**4.01**	**2.54**	**6.34**	**0.000**
High school grad	**3.55**	**2.30**	**5.49**	**0.000**
Some college/associate’s degree	**2.75**	**1.79**	**4.24**	**0.000**
College graduate	ref.	–	–	–
Family Income				
Less than $20,000	**3.55**	**2.57**	**4.91**	**0.000**
$20,000–$49,999	**1.95**	**1.44**	**2.64**	**0.000**
$50,000–$74,999	**1.56**	**1.08**	**2.26**	**0.020**
$75,000 or more	ref.	–	–	–
Population Density				
Segment in a CBSA > 1 million	0.80	0.55	1.17	0.248
Segment in a CBSA < 1 million	0.99	0.69	1.40	0.936
Segment not in a CBSA	ref.	–	–	–
Employment (past week)				
Employed full/part-time	ref.	–	–	–
Unemployed	**4.23**	**3.11**	**5.76**	**0.000**
Retired	**0.29**	**0.14**	**0.59**	**0.001**
Disabled	**4.10**	**2.88**	**5.84**	**0.000**
Other	**1.85**	**1.44**	**2.37**	**0.000**
Arrested and Booked for Breaking the Law				
No	ref.	–	–	
Yes	**7.73**	**6.18**	**9.68**	**0.000**
Overall Health Status				
Fair/Poor	**10.70**	**7.25**	**15.78**	**0.000**
Good	**6.15**	**4.17**	**9.05**	**0.000**
Very Good	**3.52**	**2.49**	**4.96**	**0.000**
Excellent	ref.	–	–	
Serious Psychological Distress in Past Year				
No	ref.	–	–	
Yes	**9.15**	**7.55**	**11.08**	**0.000**
Suicidality in Past Year				
No	ref.	–	–	
Yes	**8.14**	**6.61**	**10.04**	**0.000**
Private Health Insurance				
No	**4.14**	**3.34**	**5.14**	**0.000**
Yes	ref.	**–**	**–**	
Nicotine Dependence (past month)				
No	ref.	–	–	–
Yes	**10.46**	**8.44**	**12.96**	**0.000**
Alcohol Dependence or Abuse (past year)				
No/Unknown	ref.	–	–	–
Yes	**5.80**	**4.72**	**7.13**	**0.000**
Marijuana Dependence or Abuse (past year)				
No/Unknown	ref.	–	–	–
Yes	**12.82**	**9.33**	**17.62**	**0.000**
Cocaine Dependence or Abuse (past year)				
No/Unknown	ref.	–	–	–
Yes	**45.16**	**31.87**	**64.00**	**0.000**
Inhalant Dependence or Abuse (past year)				
No	ref.	–	–	–
Yes	**51.00**	**18.24**	**142.58**	**0.000**
Methamphetamine Dependence or Abuse (past year)				
No	ref.	–	–	–
Yes	**51.88**	**36.77**	**73.21**	**0.000**
Tranquilizer Dependence or Abuse (past year)				
No	ref.	–	–	–
Yes	**145.51**	**112.02**	**189.02**	**0.000**
Stimulant Dependence or Abuse (past year)				
No	ref.	–	–	–
Yes	**68.84**	**40.61**	**116.67**	**0.000**
Sedative Dependence or Abuse (past year)				
No	ref.	–	–	–
Yes	**67.08**	**30.53**	**147.40**	**0.000**

*Note. ref* reference group, *CI* confidence interval

#### Adjusted multivariate logistic regression models

Model 1 found that sociodemographic factors such as age, sex/gender, race/ethnicity, sexual identity, educational attainment, family income, and employment status were positively associated with opioid misuse. In Model 2, we added the socioecological factor of past criminality, which was positively associated with opioid misuse, while controlling for sociodemographic factors. In Model 3, health factors such as overall reported health, serious psychological distress in past year, suicidality in the past year, and not having private health insurance were added (while controlling for sociodemographic and socioecological factors) and were positively associated with opioid misuse. In Model 4, other substance dependence and abuse were added to the model, which was controlled for sociodemographic, socioecological, and health factors. Model 4 was selected for interpretation.

#### Comprehensive model of opioid misuse

Compared to no prior history, having past criminality was associated with significantly increased odds of opioid misuse (adjusted odds ratio [AOR] = 2.58, 95% confidence interval [CI]: 1.98–3.37, *p* < 0.001). Overall self-reported health status was associated with opioid misuse when individuals reported fair/poor (AOR = 3.71, 95% CI:2.19–6.29, *p* < 0.001), good (AOR = 3.43, 95% CI: 2.20–5.34, *p* < 0.001), and very good health (AOR = 2.75, 95% CI: 1.90–3.98, *p* < 0.001) compared to those that reported excellent health. Among individuals with no private health insurance, there was 2.12 increased adjusted odds (95% CI: 1.55–2.89, *p* < 0.001) of opioid misuse compared to participants with health insurance. Similarly, participants who experienced past serious psychological distress or suicidality had 3.05 adjusted odds (95% CI: 2.20–4.23, *p* < 0.001) and 1.58 odds (95% CI: 1.17–2.14, *p* = 0.004) of opioid misuse, respectively, when compared to those with no history. Participants exhibiting substance dependence or abuse, with the notable exception of inhalants and sedatives, were positively associated with increased adjusted odds of opioid misuse compared to those with no substance dependence or abuse (nicotine: AOR = 3.01, 95% CI: 2.30–3.93, *p* < 0.001; alcohol: AOR = 1.40, 95% CI: 1.02–1.92, *p* = 0.038; marijuana: AOR = 2.24, 95% CI: 1.40–3.58, *p* = 0.001; cocaine: AOR = 3.92, 95% CI: 2.14–7.17 *p* < 0.001; methamphetamine: AOR = 3.32, 95% CI: 1.96–5.64 *p* < 0.001; tranquilizers: AOR = 16.7, 95% CI: 9.75–28.7, *p* < 0.001; stimulants: AOR = 2.45, 95% CI: 1.03–5.87, *p* = 0.044). See Table 4 for more detail.

**Table 4:** Multivariate logistic regression examining opioid misuse and/or use disorder: 2017–2018 NSDUH

	Model 1 Sociodemographic Indicators				Model 2 Socioecological Indicator				Model 3 Health Indicators				Model 4 Other Substance Abuse or Dependence			
	AOR	95% CI		*p*-value	AOR	95% CI		*p*-value	AOR	95% CI		*p*-value	AOR	95% CI		*p*-value
		Lower	Upper			Lower	Upper			Lower	Upper			Lower	Upper	
Age																
18–25 years old	**4.06**	**1.53**	**10.77**	**0.006**	**4.08**	**1.54**	**10.85**	**0.006**	2.52	0.96	6.63	0.060	1.69	0.60	4.73	0.311
26–34 years old	**6.65**	**2.55**	**17.30**	**0.000**	**4.58**	**1.74**	**12.11**	**0.003**	**2.88**	**1.12**	**7.43**	**0.029**	2.07	0.78	5.49	0.142
35–49 years old	**4.43**	**1.71**	**11.46**	**0.003**	**2.99**	**1.14**	**7.81**	**0.026**	2.06	0.80	5.33	0.132	1.75	0.65	4.70	0.261
50–64 years old	**3.57**	**1.47**	**8.69**	**0.006**	**2.65**	**1.09**	**6.47**	**0.033**	2.30	0.95	5.55	0.065	1.90	0.73	4.92	0.182
65 years and older	ref.	–	–	–	ref.	–	–	–	ref.	–	–	–	ref.	–	–	–
Sex/Gender																
Male	**1.45**	**1.13**	**1.85**	**0.004**	1.02	0.82	1.27	0.855	1.26	1.00	1.59	0.055	1.17	0.90	1.52	0.241
Female	ref.	–	–	–	ref.	–	–	–	ref.	–	–	–	ref.	–	–	–
Race/Ethnicity																
Non-Hispanic White	**4.31**	**1.89**	**9.84**	**0.001**	**3.16**	**1.37**	**7.33**	**0.008**	**2.87**	**1.18**	**6.97**	**0.021**	2.23	0.87	5.76	0.095
Non-Hispanic Black/African American	1.90	0.72	5.00	0.189	1.40	0.53	3.74	0.493	1.46	0.52	4.09	0.463	1.47	0.49	4.42	0.482
Native American/Alaska Native	**3.87**	**1.47**	**10.19**	**0.007**	2.49	0.93	6.63	0.067	2.53	0.91	7.01	0.074	1.65	0.53	5.10	0.376
Native Hawaiian/Pacific Islander	1.66	0.31	8.88	0.547	1.55	0.30	7.98	0.592	1.47	0.27	7.95	0.647	1.06	0.19	5.75	0.949
Non-Hispanic more than one race	**4.60**	**1.67**	**12.67**	**0.004**	**2.99**	**1.04**	**8.63**	**0.043**	2.40	0.79	7.27	0.119	1.95	0.60	6.33	0.258
Hispanic	1.56	0.69	3.55	0.281	1.40	0.60	3.24	0.425	1.34	0.56	3.19	0.506	1.38	0.55	3.46	0.486
Non-Hispanic Asian	ref.	–	–	–	ref.	–	–	–	ref.	–	–	–	ref.	–	–	–
Sexual Identity																
Lesbian or gay	1.04	0.59	1.83	0.887	0.99	0.55	1.78	0.978	0.71	0.40	1.27	0.243	0.56	0.28	1.09	0.088
Bisexual	**1.96**	**1.36**	**2.81**	**0.001**	**1.75**	**1.24**	**2.48**	**0.002**	0.99	0.69	1.44	0.965	0.80	0.51	1.26	0.333
Heterosexual, i.e., straight	ref.	–	–	–	ref.	–	–	–	ref.	–	–	–	ref.	–	–	–
Educational Attainment																
Less than high school	**2.93**	**1.81**	**4.75**	**0.000**	**2.11**	**1.30**	**3.43**	**0.003**	1.58	0.97	2.57	0.067	1.21	0.71	2.07	0.484
High school grad	**2.59**	**1.65**	**4.05**	**0.000**	**1.99**	**1.28**	**3.09**	**0.003**	**1.55**	**1.00**	**2.41**	**0.049**	1.34	0.79	2.28	0.268
Some college/associate’s degree	**2.08**	**1.32**	**3.26**	**0.002**	**1.61**	**1.02**	**2.53**	**0.040**	1.28	0.82	2.02	0.272	1.19	0.71	1.99	0.496
College graduate	ref.	–	–	–	ref.	–	–	–	ref.	–	–	–	ref.	–	–	–
Population Density of Residence																
In a CBSA > 1 million	1.26	0.84	1.88	0.257	1.31	0.87	1.98	0.187	1.31	0.86	2.02	0.208	1.31	0.83	2.07	0.242
In a CBSA < 1 million	1.15	0.79	1.67	0.450	1.18	0.81	1.71	0.392	1.14	0.78	1.68	0.483	1.16	0.78	1.73	0.450
Not in a CBSA	ref.	–	–	–	ref.	–	–	–	ref.	–	–	–	ref.	–	–	–
Family Income																
Less than $20,000	**2.24**	**1.53**	**3.28**	**0.000**	**1.84**	**1.26**	**2.67**	**0.002**	1.06	0.73	1.56	0.741	0.83	0.55	1.27	0.390
$20,000–$49,999	**1.58**	**1.13**	**2.21**	**0.009**	1.40	1.00	1.96	0.051	0.93	0.67	1.30	0.679	0.85	0.59	1.22	0.374
$50,000–$74,999	1.32	0.91	1.92	0.145	1.22	0.83	1.79	0.311	1.01	0.68	1.49	0.966	0.91	0.59	1.40	0.649
$75,000 or more	ref.	–	–	–	ref.	–	–	–	ref.	–	–	–	ref.	–	–	–
Employment Status																
Employed full/part-time	ref.	–	–	–	ref.	–	–	–	ref.	–	–	–	ref.	–	–	–
Unemployed	**3.02**	**2.17**	**4.21**	**0.000**	**2.80**	**2.02**	**3.88**	**0.000**	**1.89**	**1.34**	**2.65**	**0.000**	1.47	0.99	2.16	0.054
Retired	0.71	0.28	1.84	0.478	0.73	0.28	1.88	0.509	0.60	0.24	1.47	0.256	0.62	0.24	1.63	0.324
Disabled	**2.68**	**1.67**	**4.30**	**0.000**	**2.31**	**1.42**	**3.77**	**0.001**	1.01	0.62	1.67	0.952	1.07	0.63	1.83	0.797
Other	**1.58**	**1.17**	**2.14**	**0.003**	**1.65**	**1.21**	**2.25**	**0.002**	**1.36**	**1.00**	**1.86**	**0.049**	1.24	0.86	1.79	0.252
Arrested and Booked					**5.40**	**4.26**	**6.84**	**0.000**	**4.19**	**3.34**	**5.25**	**0.000**	**2.58**	**1.98**	**3.37**	**0.000**
Overall Health																
Fair/poor									**4.58**	**2.89**	**7.26**	**0.000**	**3.71**	**2.19**	**6.29**	**0.000**
Good									**4.06**	**2.76**	**5.96**	**0.000**	**3.43**	**2.20**	**5.34**	**0.000**
Very good									**2.94**	**2.07**	**4.16**	**0.000**	**2.75**	**1.90**	**3.98**	**0.000**
Excellent									ref.	–	–	–	ref.	–	–	
No Private Health Insurance									**2.29**	**1.73**	**3.04**	**0.000**	**2.12**	**1.55**	**2.89**	**0.000**
Serious Psychological Distress ^a^									**4.20**	**3.25**	**5.44**	**0.000**	**3.05**	**2.20**	**4.23**	**0.000**
Suicidality in Past Year ^a^									**2.14**	**1.64**	**2.79**	**0.000**	**1.58**	**1.17**	**2.14**	**0.004**
Nicotine Dependence ^a^													**3.01**	**2.30**	**3.93**	**0.000**
Alcohol Dependence or Abuse ^a^													**1.40**	**1.02**	**1.92**	**0.038**
Marijuana Dependence or Abuse ^a^													**2.24**	**1.40**	**3.58**	**0.001**
Cocaine Dependence or Abuse ^a^													**3.92**	**2.14**	**7.17**	**0.000**
Inhalant Dependence or Abuse ^a^													1.80	0.23	14.23	0.571
Methamphetamine Dependence or Abuse ^a^													**3.32**	**1.96**	**5.64**	**0.000**
Tranquilizer Dependence or Abuse ^a^													**16.72**	**9.75**	**28.65**	**0.000**
Stimulant Dependence or Abuse ^a^													**2.45**	**1.03**	**5.87**	**0.044**
Sedative Dependence or Abuse ^a^													3.16	0.52	19.21	0.206

*Notes*. *ref* reference group, *AOR* adjusted odds ratio, *CI* confidence interval

^a^Compared to those not experiencing the condition

## Discussion

Opioid misuse and use disorder prevention strategies and programs must focus on multiple associated risk factors in the context of the person and their environment to ameliorate the ongoing epidemic. As epidemics do not occur in a vacuum, we accounted for the biopsychosocial characteristics associated with opioid misuse and/or use disorder. Sociodemographic, socioecological, and health factors, as well as other substance dependence or abuse were found to be independently significant for opioid misuse and/or use disorder. However, we found in our comprehensive model that socioecological indicators like criminality, health status factors including serious psychological distress and suicidality, and private health insurance were significant risk characteristics, as well as nicotine, alcohol, marijuana, cocaine, methamphetamine, tranquilizer, and stimulant dependence or abuse.

In our comprehensive biopsychosocial model we observed that sociodemographic factors functioned as controls rather than predictors for opioid misuse and/or use disorder. While other studies have focused on sociodemographic factors for describing risk in opioid misuse and overdose death [8, 9, 37, 45, 46], our model further revealed the significance of accounting for socioecological and health related risk factors in context of opioid misuse and/or use disorder. Our findings were similar to a study by Mojtabai, Amin-Esmaeili, Nejat, and Olfson [47] that found prescribed-opioid misuse was associated with criminality, mental health distress, and other substance abuse or dependence. Similarly, a study by Grigsby and Howard [34] discovered that prescription opioid and polysubstance users had the greatest probability of past-year criminality and mental health distress.

The relationship of opioid misuse and/or use disorder, mental health distress, and socioecological factors like criminality are complex, and may be co-occurring. To understand this risk process we can look to a study by Prince [22], which found that individuals with opioid misuse disorder who had a severe mental illness were at an increased risk of criminality and suicidality. The risk increased for those using only heroin, both heroin and prescription opioids, and all other substances, in that order [22]. Moreover, we found that common mental health disorders such as major depression, dysthymia, generalized anxiety disorder, or panic disorder in the general population predicted a 96% increase in prescribed opioid use [48]. While the relationship between criminality, mental health, and substance use is notable for developing tailored interventions, an overemphasis on this link may also perpetuate harmful stigma and mask important underlying factors. For example, adverse childhood experiences may contribute to all three: criminality, mental health disorders, and opioid misuse and use disorder [49–51].

Of note from our findings was that race/ethnicity in the presence of other socioecological and health factors related to polysubstance use may not be strongly associated with polysubstance dependence/abuse and opioid misuse and/or use disorder [52]. For instance, we found non-Hispanic Whites, American Indian/Alaska Natives, and non-Hispanic multiracial individuals were a significant group until polysubstance dependence/abuse was accounted for in the comprehensive model, but it may be explainable by other contextual factors [53, 54]. Whites, for example, are often prescribed more opioids compared to their Non-Hispanic Black counterparts, regardless of genuine clinical need [53]. Furthermore, other possibilities to consider between and within racial/ethnic groups are access to illicit drugs for purchase and use of drugs by friends and family members, as well as adverse childhood experiences or trauma [51, 55–57].

Other substance dependence or abuse has been associated with opioid misuse based on various risk factors [11, 25, 30, 45, 58]. In our study, we found that nicotine [25, 26], alcohol [25, 27], cocaine [58], methamphetamine [29], tranquilizers [31, 32, 59], other illicit stimulants [15], and marijuana [25] have a positive relationship with opioid misuse and use disorder. The stimulant effect from nicotine, cocaine, methamphetamine, and other illicit stimulants may mitigate the depressive effects of opioids and may increase the “high” effect [29]. Substances such as tranquilizers have been reported to be used to heighten, maintain, and extend the effect of the “high” [31–33], which may explain the elevated odds ratio of 16.7 when compared to all other substance dependence or abuse. Further research would be necessary to capture this context. Tranquilizer dependence and abuse is also of particular note, as most opioid overdose reported in the US involved some type of tranquilizer—for example, benzodiazepines [60, 61].

Our study also revealed an increased association of opioid misuse and/or use disorder with marijuana compared to non-marijuana users. This relationship, however, has been found to have mixed associations in previous studies. In the cases of marijuana dependence or abuse there is a positive relationship with opioid misuse [34]. A more recent review found that medical marijuana use may decrease the probability of opioid use [36]. Campbell et al. [36] further revealed that medical cannabis laws may slow the increase of opioid overdose deaths in states with medical cannabis laws compared to states with none. Alcohol has been another substance with mixed associations for opioid misuse and use disorder. For instance, Fernandez et al. [27] reported that alcohol dependence or abuse was not associated with opioid misuse. We found, however, in our comprehensive adjusted model that alcohol dependence or abuse was associated with a higher probability for opioid misuse, in line with the findings of Witkiewitz et al. [62]. Overall, prevention strategies and prevention programs must focus on both the combined use of legal and illicit substances.

Our study used a comprehensive approach to understand how multiple biopsychosocial characteristics relate, in context, to opioid misuse and/or use disorder. Since the current opioid crisis is not unlike prior substance use disorder crises of the past, our goal was to provide data that can be used to inform primary, secondary, and tertiary prevention efforts along the continuum from opioid misuse to use disorder—with attention to particular groups and contextual factors. By identifying risk factors within our model, we were able to contextually examine biopsychosocial characteristics to inform future research and prevention strategies to intervene upon opioid use disorder and related distal outcomes for noninstitutionalized US adults. Tailored interventions could be effective for individuals reentering society from incarceration, experiencing unemployment, suffering from psychological distress, and/or using public health insurance [63]. Examples include reentry programs, jobs placement programs, and integrated mental health and substance abuse treatment [64–67]. Nonetheless, opioid use and misuse disorder may occur alongside use of other substances, and both the determinants and effects of concurrent use must be addressed by interventions [5]. Our hope is that our results do not perpetuate stigma but rather encourage the development of effective interventions for specific populations.

Lastly, our study using a biopsychosocial model elucidated that the opioid epidemic is not an epidemic as much a syndemic. The opioid syndemic involves multiple interacting social, health, and psychological factors with comorbid substance co-use that synergizes the negative effects of opioid misuse and/or use disorder [68, 69]. Future interventions will need to acknowledge the opioid syndemic as multiple dynamic and complex factors and health outcomes that come as a result not only from misuse and/or use disorder, but policies and environmental contexts. As such, future studies will have to use complex models to move beyond one-dimensional outcomes to understand the contextual issues of opioid misuse and/or use disorder and improve not only overdose outcomes but person-level quality of life.

### Limitations

To our knowledge, this is the first US population-level study to comprehensively address risk profiles of opioid misuse using the latest national survey data available. Like most surveys of this kind, there are limitations to the NSDUH. The most prominent limitation is the use of self-reported data. These data are subject to the individual participant’s bias, truthfulness, recollection, and knowledge. Second, although the data are nationally representative, the survey is cross-sectional, and it excludes some subsets of the population. The NSDUH only targets noninstitutionalized US citizens, so active-duty military members and institutionalized groups (e.g., prisoners, hospital patients, treatment center patients, and nursing home members) are excluded. Thus, if substance use differs between US noninstitutionalized and institutionalized groups by more than 3%, data may be problematic for the total US population [44]. A particularly notable limitation of the NSDUH is that it does not include information regarding chronic pain. This omission necessarily narrowed our analysis and inhibited our ability to create a truly comprehensive model. Another issue that may have introduced bias is participant knowledge or lack thereof concerning opioids and other substances [70]. Moreover, heroin is a less commonly used opioid and there are issues in accounting for the true prevalence of this substance use [70, 71]. Finally, the opioid misuse data do not fully account for synthetic opioids like fentanyl.

## Conclusion

This study provides the most recent and comprehensive risk assessment of possible biopsychosocial characteristics indicative of opioid misuse. Findings provide the population-level risk factors to improve risk assessments and to tailor future interventions to stem and ameliorate the opioid epidemic. For instance, at-risk individuals had a history of criminality, serious psychological distress, suicidality, no private health insurance, and substance dependence or abuse. Individuals, however, are not variables representative of risk factors on an outcome to opioid misuse and/or use disorder. At a population-level analysis, we must acknowledge that results of a variable-centered approach such as this work only represent findings based on a population average. More specialized approaches, such as person-centered ones, are necessary to study specific at-risk groups and opioid misuse and/or use disorder [72]. Thus, these findings serve as a population-level risk profile using the most recent US nationally representative data to inform epidemiological trends and possible large-scale interventions.

## Data Availability

All National Survey on Drug Use and Health datasets analyzed during the current study are available in the Substance Abuse & Mental Health Data Archive (SAMHDA) database repository, https://www.datafiles.samhsa.gov/study-series/national-survey-drug-use-and-health-nsduh-nid13517, of which public access is open.

## Abbreviations

AOR: Adjusted odds ratio
CBSA: Core-based statistical areas
OR: Odds ratio
US: United States
